# The nonverbal expression of guilt in healthy adults

**DOI:** 10.1038/s41598-024-60980-0

**Published:** 2024-05-08

**Authors:** Chloe A. Stewart, Derek G. V. Mitchell, Penny A. MacDonald, Stephen H. Pasternak, Paul F. Tremblay, Elizabeth C. Finger

**Affiliations:** 1 Graduate Program in Neuroscience, Schulich School of Medicine and Dentistry, University of Western Ontario, 1151 Richmond St, London, ON N6A 3K7 Canada; 2 The Brain and Mind Institute, University of Western Ontario, London, ON N6A 5B7 Canada; 3 Department of Psychology, University of Western Ontario, London, ON N6A 5C2 Canada; 4 Department of Anatomy and Cell Biology, University of Western Ontario, London, ON N6A 5C1 Canada; 5 Department of Psychiatry, Schulich School of Medicine and Dentistry, University of Western Ontario, London, ON N6C 0A7 Canada; 6 Department of Clinical Neurological Sciences, University of Western Ontario, London, ON N6A 3K7 Canada; 7 Robarts Research Institute, Schulich School of Medicine and Dentistry, University of Western Ontario, London, ON N6A 3K7 Canada; 8 Parkwood Institute Research, Lawson Health Research Institute, London, ON N6C 2R5 Canada; 9 Graduate Program in Neuroscience, Schulich School of Medicine and Dentistry, University of Western Ontario, 1151 Richmond St, London, ON N6A 3K7 Canada

**Keywords:** Guilt, Gesture, Posture, Facial expressions, Social emotions, Psychology, Human behaviour

## Abstract

Guilt is a negative emotion elicited by realizing one has caused actual or perceived harm to another person. One of guilt’s primary functions is to signal that one is aware of the harm that was caused and regrets it, an indication that the harm will not be repeated. Verbal expressions of guilt are often deemed insufficient by observers when not accompanied by nonverbal signals such as facial expression, gesture, posture, or gaze. Some research has investigated isolated nonverbal expressions in guilt, however none to date has explored multiple nonverbal channels simultaneously. This study explored facial expression, gesture, posture, and gaze during the real-time experience of guilt when response demands are minimal. Healthy adults completed a novel task involving watching videos designed to elicit guilt, as well as comparison emotions. During the video task, participants were continuously recorded to capture nonverbal behaviour, which was then analyzed via automated facial expression software. We found that while feeling guilt, individuals engaged less in several nonverbal behaviours than they did while experiencing the comparison emotions. This may reflect the highly social aspect of guilt, suggesting that an audience is required to prompt a guilt display, or may suggest that guilt does not have clear nonverbal correlates.

## Introduction

Guilt is the emotional consequence of realizing that through one’s action or inaction one is or could be responsible for an actual harm occurring to another person^1,2^. Guilt serves two social functions: to discourage antisocial behaviour and encourage prosocial behaviours^3^. It is the prosocial action of apology and acknowledging guilt that necessitates guilt’s successful, genuine conveyance to observers^4,5^. Conveyance of sincere feelings of guilt have often been found to involve nonverbal expressions, such as kneeling or crying^6^. However, to date little research has established the specific ways in which guilt may be conveyed by the face and body.

### Nonverbal expressions of emotion

Emotion is often conveyed by language and non-language qualities of the voice, but the expression of emotion is enhanced by nonverbal behaviours, which are universal in humans, though precise expressions are culturally bound^7–9^. Patterns of nonverbal activity that signify different emotional states has attracted a great deal of scrutiny not only from the scientific community, but also from law enforcement, the media, public relations, and many others^10,11^. Four channels of nonverbal behaviour have been particularly studied in this area. The most frequently studied is facial expression, the movement of face muscles alone and in concert to create emotionally meaningful positions, such as a smile or furrowed brows^12^. Gestures involve the action of parts of the body alone or together to create or enhance emotional messages to the observer (e.g., giving a thumbs up to indicate that all is well)^13^. Closely related is posture, the position of the body or part of the body, which can convey gross emotional states, as in leaning the upper body and head away from an observer to convey discomfort^14,15^. Finally, gaze, the direction of the eyes at or away from an observer or the stimulus, can signal approach or avoidance intention^16,17^. Together, these nonverbal signals can produce a display of a person’s thoughts and emotions better than any single nonverbal signal alone^16,18^.

### Nonverbal expressions of guilt

Guilt drives reparative behaviour, that is, actions that seek to fix the harm caused, and one of the primary reparative actions is apology^19,20^. For an apology to successfully repair a harm, it must be perceived by the recipient as sincere and driven by genuine intentions to right the wrong and to not behave similarly in future^20,21^. Successfully conveying that one feels guilt has been shown to be effective in convincing the observer that the apology is authentic and backed by the intention to not cause harm again^6,22^. Observers of apologies often look to extraverbal signifiers to affirm that the guilt expressed is sincere. The failure to convey guilt or remorse nonverbally has been associated with perceptions of insincerity and manipulativeness^23,24^. Conversely, behaviours such as tears, negative facial expressions, and postural slumping have been associated with increased judgements of emotional sincerity, and more positive evaluations of the transgressor^5,6^.

Existing studies of the nonverbal expression of guilt are rare. Of the two studies that have investigated facial expression of guilt, one did not identify a unique facial expression of guilt^25^. The other identified lowering of the brow as important both for expressors of guilt and for observers to read guilt in expressors, while stretching of the lips was observed in expressors but not fundamental to observers^26^. Julle-Danière et al.^26^ also identified three gestures as key to guilt expression: touching the neck with one hand, nodding, and turning the head away. No studies have investigated posture specific to guilt; however, studies of the two related emotions of embarrassment and shame have suggested a collapsed, diminished posture, with shoulders pulled down and towards the midline of the chest and head tilted downwards^27–29^. Gaze aversion has been associated with guilt in some studies, though results are mixed^30–32^.

### The present study

Though expressing guilt is a fundamental aspect of its social purpose, no studies to date have investigated the combination of nonverbal behaviours (e.g. facial expression, gaze, gesture, and posture) associated with feeling guilty. The present study sought to fill this gap by identifying whether there is a distinct nonverbal expression associated with the real-time experience of guilt in healthy adults. We hypothesized that there would be a unique nonverbal signature of guilt that was distinct from other emotions. Based on the existing literature around guilt, as well as shame and embarrassment, we predicted that the pattern of behaviours elicited during the experience of guilt would convey submission and contrition to an observer. That is, the face would display a negative aspect, gestures would involve touching the head or neck and aversion of the head, posture would be slumped and diminished, and gaze would be averted. To this end, healthy adults took part in a video task designed to elicit guilt and comparison emotions while continuous discreet recordings were made of their faces and upper bodies. These were then analysed via automated analysis software and trained behavioural coders.

## Method

### Participant characteristics and enrollment

The sample comprised of healthy adults recruited in London, Ontario, Canada between late 2017 and early 2020 as described in Stewart et al. 2023^33^. Participants were recruited through word of mouth, as well as advertisements placed throughout the community that invited participants to take part in research on emotion. Inclusion criteria included: age 18 to 80, normal or corrected to normal vision, normal or corrected to normal hearing, and fluency in English. Exclusion criteria included current major neurological or psychological disorder, including movement disorders. All study procedures were approved by the University of Western Ontario Research Ethics Board. Participants provided written informed consent prior to undertaking study procedures and were compensated for their time.

#### Sample size calculations

Using linear regression procedures, a targeted sample size of N = 79 was retrospectively identified as sufficient to maintain a minimum power (1-ß) of 0.95 and detect a medium effect size between 0.30 and 0.36 with alpha = 0.05. Power calculations were determined using G* Power 3.1.9.7^34^ with 1 group and 10 response variables. The power calculation was based upon estimates from a similar study which detected significant group effects with effect size between ƒ^2^ = 0.30 and ƒ^2^ = 0.36 when investigating the postural and gestural expression of shame relative to pride^29^.

### Stimuli

#### Opinions and behaviour questionnaire

Participants completed a 103-item computer questionnaire on topics such as charity involvement, conservation of the environment, and identification with national identity. This questionnaire was developed by the authors based on similar questionnaires created by Statistics Canada^35^. Depending on the question, answers were given using yes/no, a scale from 1 (*not at all*) to 5 (*very much*), multiple choice, or free answer (see “supplemental material” for examples). Prior to the questionnaire, participants were informed that they would receive feedback about themselves in comparison to others based on their responses (see below).

#### Feedback statements

A short statement which purported to be derived from the above questionnaire were presented before each video. Every participant received standard feedback statements regardless of their responses to these questions (see “supplemental material”). Before undertaking the video task, participants were instructed that they would see the feedback statements, which would provide accurate feedback about themselves and their behaviours based on comparisons to prior participants and Statistics Canada. Feedback statements were developed to connect the content of the video with the past and present actions and opinions of the participant; on their own, statements did not induce their associated emotion, but supported it in combination with the video content. For example, before a video about food wastage, a participant would see “You waste much more food than average,” while a video about charitable donation would be preceded by, “You donate less than the average Canadian.” Feedback statements were either broad statements that an average person could not easily falsify about themselves, such as “You could do more to help people in Canada,” or put the subject in comparison with an average other, with the assumption that most people could not accurately know their relative place, such as “You think peacekeeping is as important as most Canadians.”

#### Video clips

Forty short video clips drawn from advertising campaigns, online home videos, films, and television shows were chosen to elicit the emotions of guilt, amusement, disgust, neutral, pride, and sadness (see “supplemental material”). Comparison emotions were selected to contrast guilt with closely related emotions (disgust and sadness), a distinct emotion (amusement), a social emotion (pride), and a baseline unemotional state (neutral). We selected 10 videos to elicit guilt, while 6 videos were chosen for each of the comparison emotions. Each clip was selected by the authors and 14 individuals (8 women, 6 men) took part in a pilot study to ensure that each clip reliably elicited its target emotion, as well as to ensure that intensity, arousal, and valence ratings were consistent across emotions (see “supplemental material”). Time windows in each video during which the emotion occurred the most strongly were also endorsed during the pilot study using CARMA video rating software, a continuous affective rating system similar to an affective rating dial^36^. Only these peak emotion windows were used in later analysis^37^. Video clip durations were between 20 s and 2 min, with an average duration of 1 min.

### Procedure

Following informed consent procedures and the collection of demographic information, participants were seated in a comfortable chair in front of a computer monitor and instructed to complete the opinions and behaviours questionnaire independently. Following questionnaire completion, the webcam was turned on and adjusted to ensure that the participant’s entire head and upper chest were visible in frame, and the participant received the full task instructions. During data collection a researcher was positioned behind a standing screen to respond to questions, concerns, or distress during the study while reducing distraction for the participant.

#### Video task

The task was programmed and run in E-Prime version 3.0 (Psychology Software Tools., Pittsburgh, PA). Participants were presented with a single feedback statement in the centre of the screen, which would remain until the participant clicked to acknowledge it. The linked video clip would then play. After the conclusion of the video, a black screen would appear and last for ten seconds, during which the participant was instructed to think about the video contents and how it made them feel. Participants were then presented with a list of 12 emotion words and asked to select the primary emotion experienced during the video clip (Table 1); only one word could be selected, and participants were instructed to choose the strongest emotion felt (endorsed emotion). This list contained the six target emotions as well as additional words potentially related to guilt (anger, contempt, embarrassment, shame), and the remaining basic emotions (fear, happiness).

**Table 1:** Emotion options presented to participants after each video.

Amusement	Embarrassment	Neutral
Anger	Fear	Pride
Contempt	Guilt	Sadness
Disgust	Happiness	Shame

**Figure 1:**
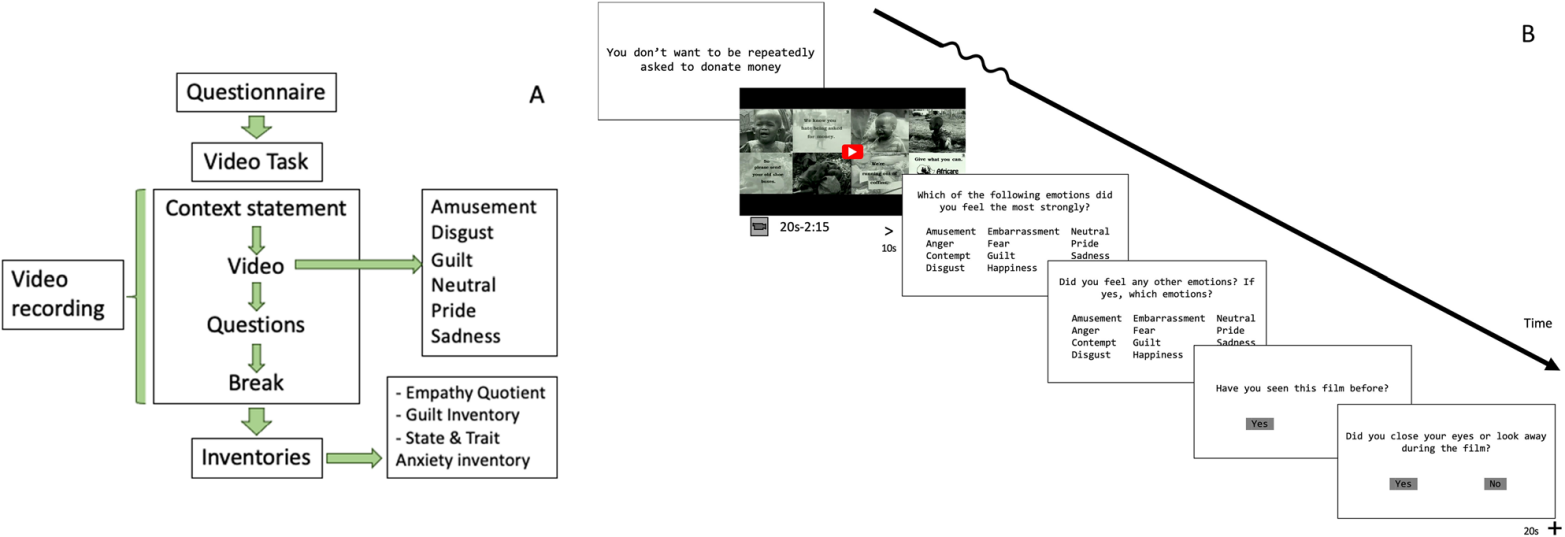
Guilt induction task. (**A**) Schematic of overall study design. (**B**) Schematic of the video trial design, depicting context statement, emotional video, and post-video questions.

After endorsing their primary emotion, participants were presented with the same list and instructed to select any additional emotions that they felt during the video clip; participants were allowed to choose as many words as they needed to describe their emotional experience, or none. This was followed by a rest period denoted by a white screen lasting 20 s. This procedure repeated in an individually randomized order until all 40 videos had been watched (Fig. 1). There were no explicit requirements for an apology or reparative action.

#### Debrief

Following the conclusion of the video task, a deception check was carried out. Participants rated whether they believed that on average the feedback statements they received were accurate and applied to them on a scale from 1 (*agree strongly*) to 5 (*disagree strongly*). Participants were then debriefed about the deception in use during the course of the study and given the opportunity to withdraw their consent to be included in the final analysis.

### Video recording, data cleaning, and analysis

The entire task was filmed with an MWay 720p webcam mounted unobtrusively atop the computer monitor on which stimuli were displayed. The recording was captured using Biopac’s AcqKnowledge linked media recording function (Biopac Systems Inc., Goleta, CA). Participants were instructed to maintain a frontward facing attitude to ensure minimal rotation and good quality recordings of facial movements. The section of peak emotion identified by the pilot study for each video (see video clips section) was clipped from the total recording using OpenShot Video Editor v2.5.1^38^ and precise timepoints provided by AcqKnowledge.

#### Action unit analysis

Recordings with a resolution of 640 × 480 at 33 frames per second were saved as MP4 files and analyzed frame by frame by FaceReader 8.1, an automated facial expression analysis software, a reliable and valid method for analyzing facial expression data (Noldus Information Technology, Wageningen, The Netherlands)^39,40^. FaceReader was set to detect each of the twenty most common facial Action Units (AUs), which were reported on a scale between 0 (not present) and 1 (maximally present). Frame by frame AU data was averaged across the entire analysis window, and scores for individual videos were averaged across all videos of the same emotion as endorsed by the participants to create a composite score for each AU for each emotion.

From the 20 available AUs produced by FaceReader, six were selected a priori based on existing studies that identified facial expressions of emotion in embarrassment, shame, and guilt, as well as related emotions such as anxiety (Table 2). AUs 12- Lip Corner Puller, 14-Dimpler, 15- Lip Corner Depressor, and 24- Lip Pressor were selected as all have been associated with the display of embarrassment and shame^30,41^. AU 4- Brow Lowerer and AU 20- Lip Stretcher are the only facial AUs that have been previously associated with the expression of guilt specifically^26^.

**Table 2:** Facial, postural, and gestural expressions with representative images, related emotions, and citations to papers that had previously identified the expression with guilt or a related emotion.

Expression	Example image	Emotions	Reference
AU 4 Brow Lowerer		Anger, Anxiety Sadness, Disgust, Confusion, Frustration, Guilt	^26,41^
AU 12 Lip Corner Puller		Happiness, Contempt, Embarrassment	^30,41^
AU 14 Dimpler		Contempt, Embarrassment	^30,41^
AU 15 Lip Corner Depressor		Sadness, Disgust, Embarrassment	^30^
AU 20 Lip Stretcher		Fear, Guilt	^26^
AU 24 Lip Pressor		Anger, Anxiety, Embarrassment	^30^
Head tilt down		Shame, Embarrassment	^30,42^
Head turn		Guilt, Embarrassment	^26,30^
Nod		Guilt	^26^
Slump/collapse upper body		Shame	^27–29^
Touch face		Embarrassment	^27^
Touch neck		Guilt	^26^

#### Postural and gestural analysis

Gestural data was scored manually using the Body Action and Posture Coding System^43^. Six body postures and gestures were chosen a priori from the 141 available postures and gestures from the Body Action and Posture Coding System based on existing research (Table 2). Lowering or tilting downwards of the head; slumping, diminishment or collapsing of the upper body; and touching of the face have all been associated with shame and embarrassment^27–30,42^. Turning of the head, nodding, and touching of the neck has been specifically associated with guilt^26^. Gestures and postures were scored by three independent raters blinded to the emotional condition of each video. Following rating of the initial 15 participants, Cohen’s kappa confirmed inter-rater reliability (k = 0.78). After retraining and consensus scoring on points of discrepancy, each rater completed postural and gestural ratings for a subset of participants so that all participant videos were completely coded. All postures and gestures were scored in 5 s increments, with postures coded as present/absent and gestures coded in terms of the number of times that they occurred. Postures were scored as percentage of time spent in the posture, while gestures were scored for number of occurrences.

#### Gaze analysis

FaceReader recorded gaze direction as forward, left, or right in relation to the screen for each video frame. Aversion of gaze has been associated in many studies with shame, embarrassment, and guilt^26,30,32,41^. Forward gaze was coded as 1 while left and right were both coded as 0. The codes were averaged across the analysis window to create a percentage of time spent looking at the screen between 0 and 100 for each video, and this percentage was averaged across all videos of the same emotion as endorsed by the participants to create a composite score for percentage of time that gaze was directed at the screen for each emotion.

### Analytic approach

All data analysis was carried out in R Studio v1.3.959^44,45^. Data for individuals who were missing single data points due to recording glitches or failures, but for whom the rest of the data was usable, were imputed using multivariate imputation by chained equations via the *mice* function *mice* package version 3.11.0^46^. All AU, gaze, postural, and gestural measures were transformed into percent of maximum possible (POMP) to account for individual variation and enable comparison between participants^47^.

To identify the variables that contributed to the distinction between emotion categories with guilt as the reference group, available AUs, gaze direction, gestures, and postures were entered into a linear mixed effects model as predictor variables using the *lmer* function in the *lme4* package v1.1–27.1^48^. To account for the repeated measures nature of the data, participant ID was entered into the model as a random effect, while gender and age were entered as fixed effects. Confidence intervals for the model were calculated using the *confint* function in the *stats* package v4.1.0^49^. Planned contrasts were carried out to investigate the specific differences between guilt and the comparison emotions for each postural, gestural, and facial variable using Quade’s ANCOVA via the *aov* and the *summary.lm* functions in the *stats* package v4.1.0^49^. All graphs were made using the *ggplot2* package v3.3.5, and in-graph calculations were performed using the *ggpubr* package v0.4.0^50,51^.

### Ethics approval

Ethics approval was awarded by the University of Western Ontario Research Ethics Board and the study was conducted in accordance with the ethical standards established in the 1964 Declaration of Helsinki and its later amendments.

### Consent to participate and publish

Informed consent was obtained from all individual participants included in the study. They specifically gave informed consent for their data to be published in peer-reviewed journals.

## Results

### Participant demographics

108 participants ranging in age from 18 to 77 (M = 39, Med = 31) participated in the study. Participants reported attending between 6 and 23 years of formal education (M = 15.963, Med = 16). Participants were excluded from the main analysis for failure to endorse feeling guilt as a primary emotion during the video task (7); technical errors in recording or analysis of video data such as videos that were too dark for the face to be clearly seen, videos in which the participant moved their face out of the frame after recording began, videos which recorded at too low of a frame rate to be analyzed, or video files which were corrupted during data transfer (20); and incomplete or absent video recordings due to power or equipment failure (4). Thus, 77 participants (36 women, 41 men) were included in the final video data analysis.

### Task debrief and deception check

No participations requested removal of their data after being debriefed. The mode and median response to the deception check of whether the participants believed the feedback statements given to them were accurate and applied to them was 2, or “Agree somewhat.”

### Endorsed emotion results

Nonverbal signal composite scores were created based on an individual’s endorsed emotion rather than the emotion targeted; thus, on an individual basis videos were reclassified to the emotion endorsed by the participant and used as part of that emotion’s composite score. Mean, range, standard deviation, and target accuracy of videos included in the composite score are reported in Table 3. Videos intended to elicit pride and guilt were most likely to trigger other emotions, while sadness and disgust were most likely to elicit their target emotion (Table 3). See “supplemental material” for further description of emotion endorsement results.

**Table 3:** Mean, range, and standard deviation for the number of emotional videos included in composite nonverbal signal scores based on individual participant endorsement of emotion, and frequency at which the target emotion of video was endorsed as the primary emotion experienced.

Emotion	M	Range	SD	Frequency of primary endorsement of target (%)
Guilt	3.99	1–9	2.02	24
Amusement	8.53	2–14	3.00	66
Disgust	4.79	0–8	1.54	76
Neutral	8.91	2–20	4.04	76
Pride	3.73	0–11	1.80	56
Sadness	7.86	2–15	2.55	82

Given the duration of the task and possibility of reduced attention that may have affected nonverbal behaviour, we analyzed the concordance between the target emotion and emotion endorsed during the first (M = 11.766, SD = 2.432) and second half (M = 12.052, SD = 3.096) of the study and did not find that they significantly differed, *t*(76) = − 0.903, *p* = 0.369.

### Video data results

#### Linear mixed effect model

Of the variables of interest, slumping or collapsing of the upper body, touching of the neck, and nodding occurred so infrequently that they could not be included in the ethogram. As such, AUs 4-Brow Lowerer, 12-Lip Corner Puller, 14-Dimpler, 15-Lip Corner Depressor, 20-Lip Stretcher, 24-Lip Pressor, tilt down of the head, turning of the head, touching of the face, and gaze direction were entered into a linear mixed effects model with both age and gender included as fixed effects in the model (Table 4). Overall, this model was statistically significant χ2(10) = 36.251 *p* < 0.001.

**Table 4:** Results of linear mixed effect model with *p* values reported for ease of interpretation. Using emotion as the outcome variable, expression of guilt as opposed to at least one of the comparison emotions was significantly predicted by level of display of 4-Brow Lowerer, 12-Lip Corner Puller, head tilt down, turning the head, and touching the face.

	Estimate	Std. Error	*t*	*p*	95% CI
4 Brow Lowerer	− 0.013	0.004	− 3.181	0.002**	− 0.021 to − 0.005
12 Lip Corner Puller	0.014	0.005	2.581	0.010*	0.003–0.024
14 Dimpler	0.003	0.012	0.242	0.809	− 0.020–0.026
15 Lip Corner Depressor	0.007	0.009	0.716	0.474	− 0.011–0.024
20 Lip Stretcher	− 0.005	0.007	− 0.669	0.504	− 0.018–0.009
24 Lip Pressor	0.007	0.005	1.384	0.167	− 0.003–0.017
Tilt Down	0.018	0.006	3.116	0.002**	0.007–0.030
Turn Head	− 0.015	0.006	− 2.495	0.013*	− 0.027 to − 0.003
Touch Face	− 0.012	0.005	− 2.281	0.023*	− 0.022 to − 0.002
Gaze	0.003	0.003	0.790	0.430	− 0.004–0.009

#### Planned contrasts

To investigate the specific relationships between these variables, planned contrasts were carried out between guilt and all comparison emotions for each of the variables identified as significant in the mixed effects model (Table 5).

**Table 5:** Summary of contrasts between guilt and comparison emotions.

	4-brow lowerer		12-Lip corner PULLER		Tilt head down		Turn head		Touch face	
	*t*	*p*	*t*	*p*	*t*	*p*	*t*	*p*	*t*	*p*
Amusement	− 0.485	0.628	7.773	< 0.001***	6.076	< 0.001***	2.730	0.006**	1.548	0.122
Disgust	2.383	0.018*	5.626	< 0.001***	8.218	< 0.001***	6.314	< 0.001***	1.428	0.154
Neutral	0.388	0.698	1.851	0.065	4.829	< 0.001***	1.948	0.052	1.267	0.206
Pride	− 1.56	0.120	1.614	0.107	5.251	< 0.001***	1.778	0.076	0.479	0.632
Sadness	0.465	0.642	2.409	0.016*	5.324	< 0.001***	3.088	0.002**	1.650	0.099

**Figure 2:**
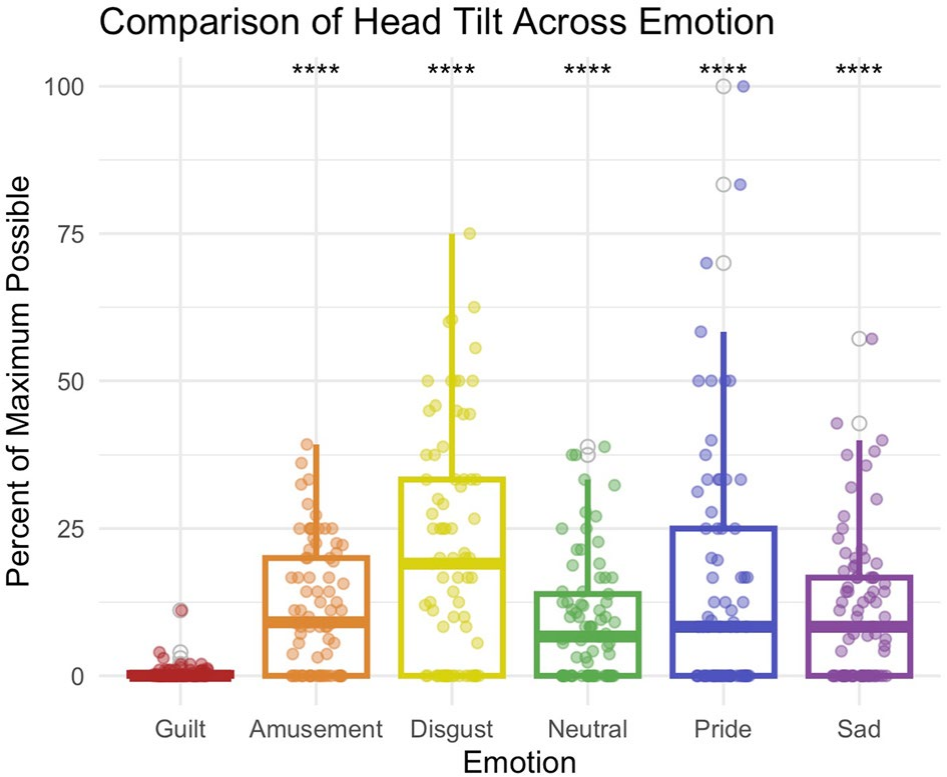
Comparison of presence of downwards head tilt across emotions. During guilt (M = 0.52), participants were less likely to tilt their heads down than in amusement (M = 11.6), *p* < 0.001, disgust (M = 21.2), *p* < 0.001, neutral (M = 9.3), *p* < 0.001, pride (M = 15.0), *p* < 0.001, or sadness (M = 11.2), *p* < 0.001.

**Figure 3:**
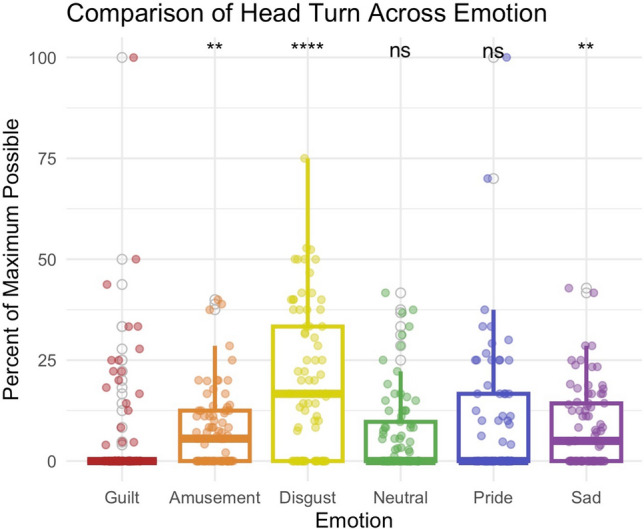
Comparison of head turning across emotions. During guilt (M = 0), participants were less likely to turn their heads away than in amusement (M = 7.7), *p* = 0.006, disgust (M = 19.1), *p* < 0.001, or sadness (M = 8.5), *p* = 0.002.

**Figure 4:**
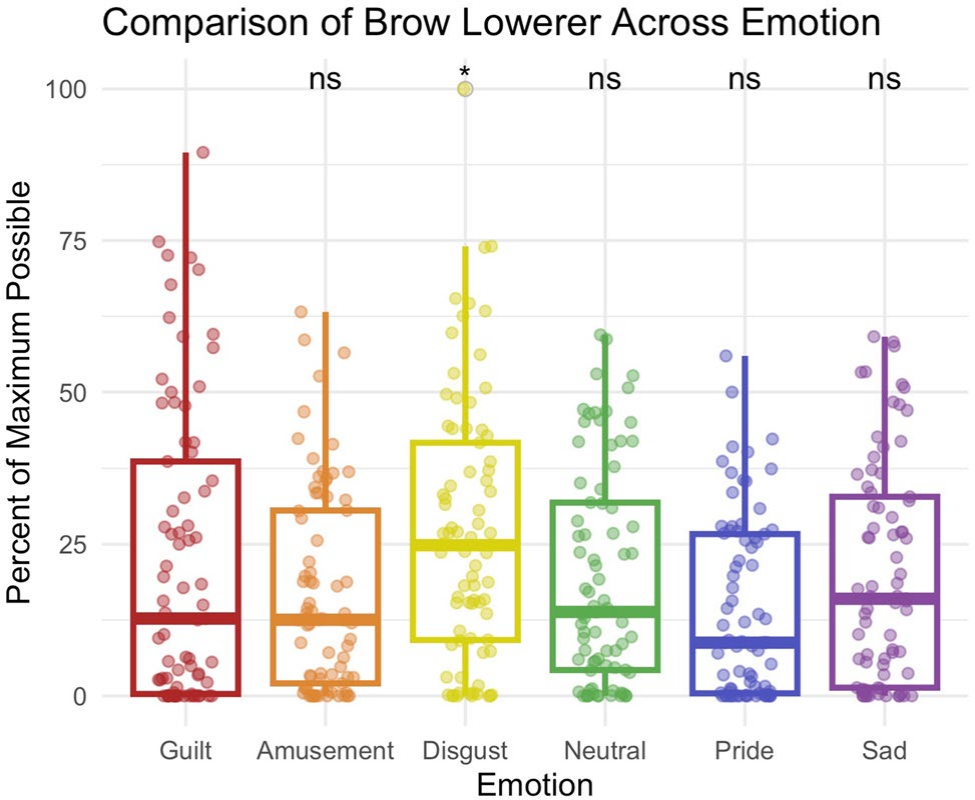
Comparison of brow lowerer across emotions. During guilt (M = 21.9), participants were less likely to lower their brow than in disgust (M = 26.9), *p* = 0.018.

**Figure 5:**
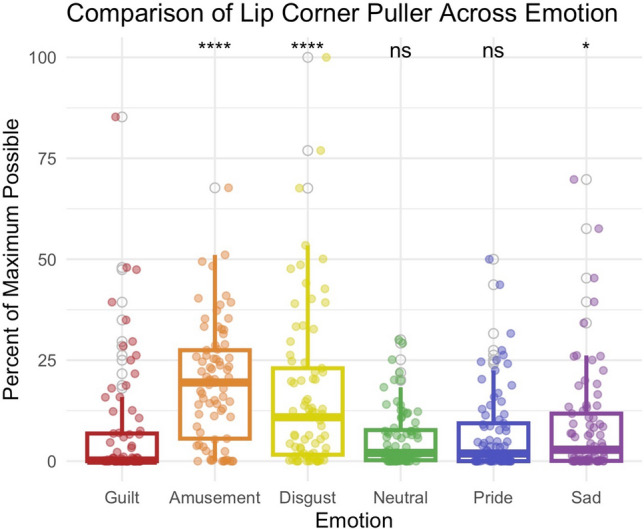
Comparison of lip corner puller across emotions. During guilt (M = 7.2), participants were less likely to display upturned lips than in amusement (M = 19.2), *p* < 0.001, disgust (M = 16.5), *p* < 0.001, or sadness (M = 8.7), *p* = 0.016.

These contrasts identified head tilt down as the most significantly different posture or gesture (*p* < 0.001 for all emotion comparisons). Participants were less likely to tilt their heads down during the guilt condition relative to any other emotion (Fig. 2). Turning of the head separated guilt from amusement, disgust, and sadness; participants were less likely to turn their heads in guilt relative to those emotions (Fig. 3). 4-Brow Lowerer (Fig. 4) was less frequent in guilt than in disgust, while 12-Lip Corner Puller (Fig. 5) was less frequent in guilt than in amusement, disgust, or sadness.

## General discussion

Emotions are commonly displayed on the face and by the body. While the expression of social emotions is often more complicated than basic emotions, there is clear evidence that social emotions can be read through the face and the body^29,52^. Though there have been some limited studies of the facial expression of guilt and related emotions, little research to date has explored the embodiment of guilt^26,30,53^. We sought to investigate the facial and bodily expressions of guilt in healthy adults, and to delineate the features that are key to the nonverbal expression of guilt. This study has provided the first combined exploration of postures, gestures, gaze, and facial expression in guilt without direct social interaction.

We identified differences between guilt and the comparison emotions on several facial, postural, and gestural variables. Head tilt down, turning the head, Lip Corner Puller, and Brow Lowerer were found to be significantly different in guilt relative to at least one other emotion. While touching of the face was identified by the omnibus test as a potential feature of guilt, these differences did not survive in the follow-up contrasts. The other facial expression variables under consideration, AUs Dimpler, Lip Corner Depressor, Lip Stretcher, and Lip Pressor did not contribute significantly to the ethogram. Direction of gaze also did not contribute significantly.

Both Brow Lowerer and Lip Corner Puller were less commonly observed in guilt than several comparison emotions. Brow Lowerer initiates a furrowed brow common in negative emotions like disgust, sadness, or frustration^54,55^. This AU was engaged during guilt about the same proportion of time as it was during all other emotions except disgust, where it was engaged more. This difference may indicate a difference in the expression of disgust, or the intensity of disgust felt, rather than a particular difference with guilt, which is borne out by the comparison of Brow Lowerer to other emotions (see “supplemental material”). Lip Corner Puller pulls the corners of the lips up and out, and is often seen as an aspect of the appeasement display when an embarrassed individual attempts to lighten the experience via an embarrassed smile^30,53,56^. This facial display was less common in guilt compared to amusement, disgust, and sadness. In amusement, this is likely reflective of smiling or laughter, while in disgust this relative increase in the activation of Lip Corner Puller compared to guilt may represent grimacing, nervous laughter or smiling, genuine amusement at disgusting stimuli, or an attempt to distance the self from distressing feelings^57–59^. This is likely again to be reflective of a difference of those emotions, rather than a distinguishing feature of guilt. However, it is interesting to note that individuals did not engage in these potentially coping-related behaviours in the negative experience of guilt while they did in disgust. Participants were less likely to tilt their heads down in guilt than in any other emotion. This finding was unexpected and in contrast to previous studies, as downwards-facing head posture has been found to be one of the most common aspects of the display of embarrassment and shame; this may reflect the absence of an audience^30^. Similarly, turning the head away was significantly less common in guilt compared to amusement, disgust, and sadness, with similar trends in comparison to neutral and pride, despite being previously identified as key for guilt in a feigned wrongdoing scenario^26^. For amusement, turning away may be secondary to movement of the head during laughter, though the exact reason for this difference is unclear. In disgust and sadness, this gesture may reflect evasion, as participants were allowed to turn away from stimuli that distressed them, which is a common reaction to objects of disgust or sadness^60^. Again, it is notable that this evasive move was not observed in guilt.

There are a few possible explanations for the unexpected findings in this study. One possibility is that these findings are reflective of the social nature of guilt. Both tilting and turning of the head were less common in guilt relative to other emotions, while aversion of gaze was consistent between guilt and the comparison emotions. These gestures have previously been strongly associated with the display of guilt in tasks that involved in-person interaction^26,32,53^. The absence of these gestures in this study may indicate that the commission of these movements is a purely social gesture, that is, they make up an appeasement or evasion display intended to enhance an apology, offer submission or remorse, evade the emotional consequences of looking into a victim’s eyes, or some other social motive. Absent a victim or observer who is aware of the performer’s guilt, as in this task, the drive to immediately enact these behaviours disappears. This may also hold true for the lack of engagement of facial AUs. While it is difficult to avoid crumpling one’s face when disgusted, to control a smile while laughing, or to control the raising of the brow during surprise, our results suggest there may be no instinctual drive to make a guilty face in the absence of an observer to acknowledge and attempt to ameliorate one’s guilt to.

Another possibility is that the relatively level, forward facing, direct posture of the head and direct gaze reflects attentional capture by the guilt-inducing stimuli^61^. Often, attention is captured by arousing stimuli, even if the arousing stimulus is negative in nature^62^. The guilt stimuli were also rendered personal by the context statements presented at the beginning of each video, which directly related the content of the video to the participant. While this was common across all film clips, the guilt film clips in particular were the most self-focused, as they were intended to directly appeal to the viewer’s actions, behaviours, or opinions. Thus, while some videos in other emotion categories addressed themselves to the viewer (to appeal to the individual to purchase something, to avoid certain behaviours, etc.), a majority of the guilt videos specifically addressed the participant and invited them to reflect upon themselves. Previous research has consistently found that attention is easily captured and held by self-focused stimuli^63,64^. As the guilt videos remained consistently personal throughout, they may have directed attention more strongly to the video than other emotions.

### Limitations

One potential limitation of this study is that there was no measurement of emotional intensity or arousal taken on a per-video basis. Therefore, it is not possible to know if the observed changes in the facial, postural, gestural, or gaze variables are reflective of a difference in emotional expression, of emotional intensity, of the general arousal level, or some combination thereof. It is also not possible to explore whether there is a parametric correlation between the intensity of the emotional experience and the intensity of nonverbal expressions. This is especially important as past research has suggested that guilt expressions particularly depend on the intensity of the underlying guilt feelings^26^. Arousal and intensity measurements should be collected during or after each individual video to better characterize the emotional experience underlying the observed expressions. Another limitation is that the arithmetic mean of each variable was taken throughout the entire analysis window and averaged across all videos of that emotion type. This creates a conservative estimate of many nonverbal variables, as the predominant emotion, along with its consistency and intensity, may vary considerably throughout the course of even a relatively short video^65^. More granular epochs, or more continuous examination of the nonverbal signals may enable the capture of subtle expressional elements that may otherwise be lost to averaging. Another possible limitation is the lack of specific exclusion criterion to prevent individuals with facial muscle disorders from taking part in the study, which could have affected the facial expression results. Although no patients with obvious facial weakness were observed, stricter exclusion criteria would prevent a similar issue in future studies. A further limitation is the lack of an explicit social aspect to this study. As previous studies have shown that having an audience can impact nonverbal displays of emotion, the lack of an audience may have limited the usefulness of this paradigm to elicit nonverbal guilt. Future studies could include a social observation aspect to account for this. The relatively low rate of endorsement of guilt for each stimulus reflects a challenge of this method for guilt elicitation. The videos were a standard set that did not specifically target areas of guilt for individuals, and instead relied on broader appeals to typical areas of guilt (e.g. climate change, charity). While attempts were made to personalize the content with feedback statements, more personalized stimuli would likely be able to elicit guilt more consistently. To achieve higher rates of guilt endorsement, future studies could consider tailoring stimuli based on participants’ interests or use another method to elicit guilt such as feigning wrongdoing. There are potential weaknesses in the usage of the Facial Action Coding System (FACS), particularly automated systems as was used in this study. Because of the reliance on an algorithm to generate the AUs, elements of the videos themselves, such as quality, lighting, colour, and angle, as well as of the individual in the video, such as hair length or position, glasses, piercings, or head scarves, can all greatly impact the ability of the system to correctly classify AUs^66,67^. Similarly, studies have found that the underlying algorithm has not been well trained on non-Western faces, meaning that the system does more poorly when attempting to analyze racialized groups^68,69^. All efforts were made in the present study to ensure that video quality was sufficient and that participants were well placed with minimal facial obstructions, leading to a fairly high rate of rejection of videos for analysis. Nonetheless, it is possible that video or participant qualities may have affected the video analysis, and thus the results of this study must be interpreted with caution. Future studies should take care to ensure that video and participant qualities are sufficient to be coded using an automated system, and to use systems which are trained on more diverse training sets.

### Conclusion

This study sought to address the current gap in the literature around the nonverbal expression of guilt in healthy adults. We found an unexpected pattern of under-reactivity to guilt relative to other emotions that is potentially reflective of guilt’s social nature, the absence of reflexivity or a hardwired expression for guilt, its capacity to capture and hold attention, or some combination thereof. These findings suggest directions for future studies to address the lack of knowledge surrounding the nonverbal expression of guilt. In particular, future studies may include a social aspect to the paradigm, such as including an audience for some participants but not others, or by having an observer say the feedback statements to participants, to expand on the possibility of guilt expression being bound to social pressures. It would be particularly valuable to investigate the downwards head tilt in a social version of the paradigm, as it has previously been identified as a relevant signal but was much less likely to be observed during guilt in this study.Additional future directions include explorations of nonverbal guilt expression in children who are developing guilt, or in populations outside of the North American context who might have different conceptualizations and experiences of guilt, or culturally bound expressions of guilt.

### Supplementary Information

Supplementary Tables.

## Data Availability

Data and supplementary information are uploaded on the Open Science Framework, an open science platform. Please use this view-only link to access this material on OSF: https://osf.io/kvb3y/?view_only=d9a0993561244217aff5a072c70505ff. This study’s design and its analysis were not pre-registered.
