# Supplementary material for: The nonverbal expression of guilt in healthy adults
Source: Sci Rep. 2024 May 8;14:10607. doi: 10.1038/s41598-024-60980-0 (PMC11078964; doi:10.1038/s41598-024-60980-0)
Supplement: Supplementary file 1 — Supplementary Tables. [file 41598_2024_60980_MOESM1_ESM.pdf]

The nonverbal expression of guilt in healthy adults

Chloe A Stewart, Derek GV Mitchell, Penny A. MacDonald, Stephen H Pasternak, Paul F Tremblay, Elizabeth C Finger

Table of Contents

Questions and response options for Opinions and Behaviour Questionnaire 3
Supplemental Table 1. Video clip details ... 15
Supplemental Table 2. Results of video pilot study. ... 17
Supplemental Table 3. Video clips ... 20
Supplemental Table 4. Feedback Statements ... 21
Supplemental Table 5. Means, standard deviations, and ranges for trait and state characteristics. ... 22
Supplemental Table 6. Total counts of primary emotions endorsed by target emotion ... 23
Supplemental Table 7. Total numbers of primary emotions endorsed and congruence with the video's target emotion, with total emotions broken down by valence and social nature ... 23
Supplemental Table 8. Counts of secondary emotions endorsed by target emotion ... 24
Supplemental Table 9. Total numbers of secondary emotions endorsed, with total emotions broken down by valence and social nature ... 24
Supplemental Table 10. Results of paired Wilcoxon tests for AU 4-Brow Lowerer ... 25
Supplemental Table 11. Results of paired Wilcoxon tests for AU 12-Lip corner puller ... 25
Supplemental Table 12. Results of paired Wilcoxon tests for head tilt down ... 25
Supplemental Table 13. Results of paired Wilcoxon tests for head turn ... 25
Supplemental Table 14. Results of paired Wilcoxon tests for face touch ... 26

Questions and response options for Opinions and Behaviour Questionnaire

Do you think that it is important to help the poor or underprivileged?

1. Yes
2. No
3. Don't know

Do you think that you personally could do more to help the poor or underprivileged here in Canada?

1. Yes
2. No
3. Don't know

Do you think that other people in your age group could do more to help the poor or underprivileged in Canada?

1. Yes
2. No
3. Don't know

In the past 12 months, have you done any unpaid work for any charitable organization?

1. Yes
    a. If yes, how many organizations? ______
    b. How much time on average per week, in hours? _____
2. No

In the past 12 months, have you made any charitable donations of money, or raised money for a charitable organization?

1. Yes
    a. If yes, how many organizations? _____
    b. How much money on average per month? _____
2. No

In the past 12 months, have you made any donations of items or food to a charitable organization?

1. Yes
    a. If yes, how many organizations? _____
    b. How many donations have you made on average per month? _____
2. No

On a scale from 1 to 5, how often do you think about volunteering or giving money, but ultimately decide not to?

1	2	3	4	5
Not very often				Very often

On a scale from 1 to 5, how often to you give spare change to charitable causes asking for money in public places?

1	2	3	4	5
Never				Always

On a scale from 1 to 5, how important do you think small donations of spare change are to charities?

1	2	3	4	5
Not important at all				Very important

Do you think that you personally could do more to help the poor or underprivileged abroad?

1. Yes
2. No
3. Don’t know

Do you think that other people in your age group could do more to help the poor or underprivileged abroad?

1. Yes
2. No
3. Don’t know

On a scale from 1 to 5, how important is it to you to support individuals in other countries who are poor or underprivileged?

1	2	3	4	5
Not important at all				Very important

Do you find constant requests for money or time from charitable organizations to be exhausting or irritating?

1. Yes
2. Sometimes
3. No

On a scale from 1 to 5, how exhausting or irritating do you find charity appeals?

1	2	3	4	5
Not exhausting or irritating at all				Very exhausting and irritating

On a scale from 1 to 5, how often do you change the channel or ignore a commercial when it becomes clear that it is a charity appeal?

1	2	3	4	5
Never				Always

On a scale from 1 to 5, how often do you wish that you did not have to see charity appeals, whether they are TV commercials, print ads, or some other appeal?

1	2	3	4	5

Never				Always

On a scale from 1 to 5, how important is it to you to protect or defend individuals fleeing war or unrest in their home countries?

1	2	3	4	5
Not important at all				Very important

On a scale from 1 to 5, how supportive are you of Canada's current efforts to resettle refugees in Canada?

1	2	3	4	5
Not at all supportive				Very supportive

In the past 12 months have you been involved, through donations of money, time, or goods, in efforts to resettle refugees in Canada?

1. Yes
2. No

Environment and Conservation

In the past 12 months have you engaged in formal or informal unpaid activities aimed at conservation or preservation of the environment or wildlife?

1. Yes
    a. How much time on average per week, in hours? _____
2. No
3. Don't know

In the past 12 months did you personally contribute to, or were you an unpaid member of, a group or club whose primary focus was environmental protection or preservation?

1. Yes
    a. If yes, how many organizations? ______
2. No
3. Don't know

Which statement best describes your attitude towards recycling?

1. I recycle all recyclable materials regularly at home and in public, regardless of inconvenience
2. I recycle most recyclable materials regularly at home and in public, if there is a receptacle nearby
3. I occasionally recycle recyclable materials at home and in public
4. I rarely recycle recyclable materials at home or in public
5. I never recycle recyclable materials at home or in public

Do you have a washing machine in your house?

1. Yes
2. No

How frequently do you (or the person responsible in your home) do laundry, per month?

1. 1-2 times
2. 2-4 times
3. 4 times or more

What temperature of water do you usually use to wash your laundry?

1. Hot
2. Warm
3. Cold
4. Don't know

Do you usually use a dryer, or do you air dry clothing where possible?

1. Usually use a dryer in both summer and winter
2. Usually use a dryer in winter, but not in summer
3. Do not have/do not use a dryer

How often do you use the stove or oven to prepare meals?

1. Less than once a week
2. For about ¼ of all meals
3. For about half of all meals
4. For about ¾ of all meals
5. For all or almost all meals

On average, how often do you bake desserts (cakes, cookies, etc.)?

1. Less than once a week
2. 1-2 times a week
3. 3-4 times a week
4. 5-6 times a week
5. 6-7 times a week
6. More than 6-7 times a week

On average, how often do you bake non-desserts (breads, muffins, etc.)?

1. Less than once a week
2. 1-2 times a week
3. 3-4 times a week
4. 5-6 times a week
5. 6-7 times a week
6. More than 6-7 times a week

How frequently do you vacuum your whole home?

1. Less than once a week
2. 1-2 times a week
3. 3-4 times a week
4. 5-6 times a week
5. 6-7 times a week
6. More than 6-7 times a week

How many personal computers do you use regularly? ____
On average, how many hours do you have your main personal computer turned on per week? ____
On average, how many hours do you use your main personal computer per week? ____
On the average day, for how many hours are the lights in your house left on? ____
On a scale from 1 to 5, how important is preserving the environment to you?

1	2	3	4	5
Not important at all				Very important

In the past 12 months, what percentage of your purchases were of environmentally friendly or “green” products?

1. Less than 10%
2. 10-20%
3. 20-30%
4. 30-40%
5. 40%-50%
6. 50% or more

On a scale from 1 to 5, how important is sustainable farming/food production to you?

1	2	3	4	5
Not important at all				Very important

Health and Safety

Are you able to drive?

1. Yes
2. No

Do you currently own or regularly operate a car?

1. Yes
2. No

In the past 12 months, have you sped while driving, passed without adequate space, or otherwise driven unsafely?

1. Yes, frequently
2. Yes, on a few occasions
3. Yes, rarely
4. No

In the past twelve months have you donated blood to the Canadian Blood Services?

1. Yes
2. No
3. I am unable to do so due to a medical disorder or legislated restrictions.

Are you currently registered as an organ donor, or would you be willing to register to donate tissue or organs?

1. Yes
2. No
3. I am unable to do so due to a medical disorder or legislated restrictions.

On a scale from 1 to 5, how important is blood and organ donation to you?

1	2	3	4	5
Not important at all				Very important

On a scale from 1 to 5, how easily do you get sad when watching a sad movie or thinking about something sad?

1	2	3	4	5
Not easily at all				Very easily

In the past 12 months have you experienced bereavement due to the death of a close loved one?

1. Yes
2. No

In the past 12 months have you experienced bereavement due to the death of a pet?

1. Yes
2. No

Have you or someone close to you ever been diagnosed with cancer?

1. Yes
2. No

On a scale from 1 to 5, how important is finding a cure for cancer to you?

1	2	3	4	5
Not important at all				Very important

Do you donate time or money to charities or groups dedicated to curing cancer?

1. Yes
    a. If yes, how many organizations? ______
    b. If you donate time, how much time on average per week, in hours? _____
    c. If you donate money, how much money on average per month? _____
2. No

On a scale from 1 to 5, how often does seeing people crying make you feel sad?

1	2	3	4	5
Never				Always

Have you or someone close to you been diagnosed with a dementia such as Alzheimer's disease?

1. Yes
2. No

On a scale from 1 to 5, how often do you cry, or feel like crying, while watching a sad movie or thinking about something sad?

1	2	3	4	5
Almost never				Almost always

On a scale from 1 to 5, how strongly do you feel sorry for people whose parent(s) has recently died?

1	2	3	4	5
Not very strongly				Very strongly

On a scale from 1 to 5, how strongly do you feel sorry for people whose child(ren) has recently died?

1	2	3	4	5
Not very strongly				Very strongly

On a scale from 1 to 5, how strongly do you feel sorry for people who have recently lost a pet?

1	2	3	4	5
Not very strongly				Very strongly

On a scale from 1 to 5, how likely are you to feel sick to your stomach if you see someone vomiting?

1	2	3	4	5
Never				Always

On a scale from 1 to 5, how much does it bother you to see food that has been contaminated in some way, such as food that is rotting or food that has bugs in it?

1	2	3	4	5
It doesn't bother me at all				It bothers me a great deal

Would you rather throw food out than risk eating something that has spoiled?

1. Yes
2. No

On average per month, what percentage of the food you purchase do you throw away because it has gone bad or you fear it has gone bad?

1. Less than 10%
2. 10-20%
3. 20-30%
4. 30-40%
5. 40%-50%
6. 50% or more

How frequently per month do you forget to eat something, allowing it to go bad?

1. Less than 10%
2. 10-20%
3. 20-30%
4. 30-40%
5. 40%-50%
6. 50% or more

On a scale from 1 to 5, how important is preventing food waste to you?

1	2	3	4	5
Not important at all				Very important

On a scale from 1 to 5, how important is it to you to ensure that you and your home are tidy and free of rot, mold, or bugs?

1	2	3	4	5
Not important at all				Very important

On a scale from 1 to 5, how sick do you feel when you see a bug or maggot in or near your food?

1	2	3	4	5
Not sick at all				Very sick

On a scale from 1 to 5, how likely would you be to eat food that had been contaminated with someone else's pus or another bodily fluid?

1	2	3	4	5
Not likely at all				Very likely

On a scale from 1 to 5, how anxious do you feel at the idea of vomiting?

1	2	3	4	5
Not anxious at all				Very anxious

On a scale from 1 to 5, how often do you suffer from nausea or discomfort while travelling as a passenger in a vehicle such as a car or boat?

1	2	3	4	5
Never				Always

Have you ever avoided taking a journey because you were worried about becoming nauseas or throwing up?

1. Yes
2. No

Do you currently have at least one child?

1. Yes
2. No
    a. Would you like to have a child at some point in the future?
        i. Yes

ii. No
iii. Not sure yet

Would you ever consider adopting a child from foster care?
1. Yes, I currently have one or more adopted children
2. Yes, I would like to one day adopt a child
3. Yes, I would consider it in the future, when I am older or more stable
4. No, I have considered it and do not think it is right for me
5. No, I have never considered it and would not be willing to adopt a child

On a scale from 1 to 5, how important do you think adoption and foster care are for society?

1	2	3	4	5
Not important at all				Very important

Advertisement and Consumption

Do you find that you laugh more easily than most people you know?
1. I laugh less easily
2. I laugh about as easily
3. I laugh more easily

On a scale from 1 to 5, how often do you laugh when you are with friends?

1	2	3	4	5
Very rarely				Very often

On a scale from 1 to 5, how much do you enjoy laughing?

1	2	3	4	5
Not at all				Very much

On a scale from 1 to 5, how influential is a celebrity endorsement on your decision to buy a product?

1	2	3	4	5
Not influential at all				Very influential

Have you ever purchased something because your favourite celebrity endorsed it?
1. Yes
   a. Did you subsequently regret that purchase?
      i. Yes
      ii. No
2. No

How many languages do you speak?
1. 1-2
2. 2-4
3. 4 or more

Have you ever considered learning another language?

1. Yes
    a. Are you currently learning another language?
        i. Yes
        ii. No
2. No

On a scale from 1 to 5, how much influence do you think advertisements have on your buying behaviour?

1	2	3	4	5
Not influential at all				Very influential

Have you ever purchased something just because you liked the ads?

1. Yes
    a. Did you subsequently regret that purchase?
        i. Yes
        ii. No
2. No

On a scale from 1 to 5, how important is humour in advertising to you?

1	2	3	4	5
Not important at all				Very important

On a scale from 1 to 5, how memorable do you find funny ads?

1	2	3	4	5
Not memorable at all				Very memorable

Have you ever chosen one product over another just because their ad was funnier?

1. Yes
2. No

On a scale from 1 to 5, how important is it to you to have the newest and latest technology?

1	2	3	4	5
Not important at all				Very important

Do you enjoy receiving or purchasing new things?

1. Yes
2. No

Do you think that having new things is important?

1. Yes
2. No

National Identity

Where were you born?
1. In Canada
2. Outside Canada

What is your current citizenship status?
1. Canadian by birth
2. Landed immigrant
3. Refugee
4. Currently seeking to become a Canadian citizen
5. Not currently seeking to become a Canadian citizen

On a scale from 1 to 5, how proud are you of being Canadian?

1	2	3	4	5
Not proud at all				Very proud

On a scale from 1 to 5, how important is your Canadian identity to you?

1	2	3	4	5
Not important at all				Very important

On a scale from 1 to 5, how important is it to you to carry on Canadian traditions, customs, and holidays?

1	2	3	4	5
Not important at all				Very important

On a scale from 1 to 5, how important is it to you to know about Canadian history?

1	2	3	4	5
Not important at all				Very important

Do you support the Canadian government's efforts to celebrate Canadian history?
1. Yes
2. No

On a scale from 1 to 5, how strong is your sense of belonging to Canada?

1	2	3	4	5
Very weak				Very strong

On a scale from 1 to 5, how strong is your sense of belonging to the world?

1	2	3	4	5
Very weak				Very strong

Do you feel connected to humanity?
1. Yes
2. No

On a scale from 1 to 5, how important is it to you to be a good Canadian?

1	2	3	4	5
Not important at all				Very important

Do you typically think of yourself and your fellow Canadians as generally good, kind, and polite?

1. Yes
2. No
3. Don't know

On a scale from 1 to 5, how much do you enjoy watching the Olympics?

1	2	3	4	5
Not at all				Very much

On a scale from 1 to 5, how proud of being a Canadian do you feel when Canada does well in the Olympics?

1	2	3	4	5
Not proud at all				Very proud

On a scale from 1 to 5, how proud do you feel of humanity when you see other people succeeding?

1	2	3	4	5
Not proud at all				Very proud

Are you, or is at least one immediate family member, a current or veteran member of the Canadian Armed Forces?

1. Yes, I am in the armed forces, or a veteran
2. Yes, an immediate family member is in the armed forces or a veteran
3. Yes, both I and an immediate family member are in the armed forces or a veteran
4. No, neither I nor an immediate family member are in the armed forces or a veteran

On a scale from 1 to 5, how important is Canada's dedication to peacekeeping to you?

1	2	3	4	5
Not important at all				Very important

On a scale from 1 to 5, how proud are you of the Canadian Armed Forces and their contribution to Canada's security?

1	2	3	4	5
Not proud at all				Very proud

On a scale from 1 to 5, how important is it to you to remember Canada's contributions in wartime?

1	2	3	4	5
Not important at all				Very important

Supplemental Table 1. Video clip details

Video Name	Target Emotion	Length	Analyzed Length*	Topic	Source Agency/Film	Source Country
**60 Seconds Blood Donation**	Guilt	60s	45s	Blood Donation	Canadian Blood Services	Canada
**Africare - Shoeboxes**	Guilt	30s	20s	Poverty/Abroad	Africare	USA
**Black Friday**	Guilt	30s	8s	Poverty/Canada	Salvation Army	Canada
**WWF Laptop**	Guilt	2m15s	1m20s	Environment/Climate Change	World Wildlife Fund	USA
**WWF T-Shirt**	Guilt	1m50s	1m30s	Environment/Climate Change	World Wildlife Fund	USA
**Don't Almost Give-Jack**	Guilt	30s	20s	Poverty/Canada	Ad Council	USA
**Let Them Figure It Out**	Guilt	60s	53s	Environment/Climate Change	Government of Ontario	Canada
**One Child**	Guilt	1m40s	1m20s	Poverty/Abroad	Save the Children	UK
**Strawberry Wasted**	Guilt	60s	15s	Food Waste	Ad Council	USA
**Without...**	Guilt	1m15s	57s	Poverty/Abroad	Save the Children	USA
**Go First**	Amusement	30s	18s	Advertisement	Cars.com	USA
**Dad Makes Cookies**	Amusement	30s	21s	Adoption	Ad Council	USA
**Charlie Sheen**	Amusement	30s	20s	Advertisement	DirecTV	USA
**French**	Amusement	45s	36s	Adoption	Ad Council	USA
**Haircut**	Amusement	30s	20s	Adoption	Ad Council	USA
**Sinking**	Amusement	40s	30s	Advertisement	Berlitz	USA
**Drinking 6000 Blended Maggots**	Disgust	45s	40s	Insect consumption, vomiting	Where's My Challenge	UK
**Custard**	Disgust	60s	45s	Food contamination	Braindead	New Zealand
**The Poop Lady**	Disgust	55s	47s	Food contamination	Hoarders	USA
**Rotting Chicken**	Disgust	1m16s	1m12s	Rotting food	Home video/ David0101010	Unknown

**Rotting Meat**	Disgust	1m36s	1m31s	Rotting food	Home video/ agnozja	Unknown
**Toxic Food Environment**	Disgust	20s	17s	Vomiting	Supersize Me	USA
**Cotton**	Neutral	1m	30s	Informational	How It's Made	Canada
**Erasers**	Neutral	45s	23s	Informational	How It's Made	Canada
**Fibre Optics**	Neutral	40s	20s	Informational	How It's Made	Canada
**Playing Cards**	Neutral	35s	18s	Informational	How It's Made	Canada
**Rubber Gloves**	Neutral	40s	20s	Informational	How It's Made	Canada
**Stickers**	Neutral	35s	18s	Informational	How It's Made	Canada
**Fly the Flag**	Pride	60s	52s	Canadian identity	Air Canada	Canada
**Greatest Human Achievements**	Pride	1m55s	1m30s	World unity/human excellence	Questar Video	UK
**I Am Canadian**	Pride	1m	39s	Canadian identity	Molson Canadian	Canada
**Made From Canada**	Pride	45s	37s	Canadian identity	Molson Canadian	Canada
**Peacekeeping**	Pride	1m10s	53s	Canadian Peacekeeping	Royal Canadian Mounted Police	Canada
**The Anthem**	Pride	1m30s	1m18s	World unity	Samsung Mobile	USA
**12 Days of Christmas**	Sadness	1m30s	1m9s	Anti-drunk driving	Transport Accident Commission	Australia
**Dying Wife**	Sadness	1m20s	1m15s	Death of a loved one	Home video/ Erin Solari	USA
**Madly in love**	Sadness	1m45s	1m10s	Loss of a loved one to dementia	Human Kind	USA
**Last Minutes with Oden**	Sadness	1m10s	59s	Death of a pet	Phos Pictures	USA
**Notification**	Sadness	45s	25s	Death of a loved one	The Messenger	USA
**SickKids vs Cancer**	Sadness	40s	19s	Childhood cancer	SickKids	Canada

*Analyzed length indicates the time window of analysis which began at average onset of emotion experienced based on piloting and ended at the conclusion of the video clip. For example, for a 60s second video clip with an analyzed length of 45s, the time window analyzed began 15 seconds after the start of the clip and continued until the video ended.

Supplemental Table 2. Results of video pilot study. The negative-positive axis, calm-aroused axis, and dominated-dominating axis were rated from 1-9, with 1 representing very negative, calm, and dominated and 9 representing very positive, aroused, and dominating. Emotions are presented with the percentage of participants who endorsed that emotion as being their primary emotion felt. Participants were also allowed to freely generate any additional emotions they experienced.

Video	Emotion Target	Neg/ Pos	Calm/ Aroused	Dom/ Dom	Emotion Endorsed	Other Emotion Endorsed
**Sinking**	Amusement	7.3	5.4	5.8	Amusement(100)	Happiness, laughter, pride
**Go First**	Amusement	6.2	3.9	5.1	Amusement(85), Neutral(15)	Neutral, amusement, humour
**Haircut**	Amusement	6.2	3.9	5.4	Amusement (67), Neutral(22), Happy(11)	Humour, happy
**Charlie Sheen**	Amusement	5.6	3.4	5.1	Amusement(75), Neutral(25)	Humour
**Dad Makes Cookies**	Amusement	6.4	3.5	5.3	Amusement (64), Neutral (22), Pride(7), Happy (7)	Confusion, Amusement, Excitement, Satisfaction, Pride
**French**	Amusement	6.4	3.8	5.4	Amusement (64), Neutral (29), Happy(7)	Happy, pride
**Drinking 6000 Blended Live Maggots**	Disgust	3.1	5.6	3.6	Disgust(70), Amusement(8), Sadness(8), Neutral(7), Anger(7)	Disgust, amused
**Poop Lady**	Disgust	2.5	5.8	4.8	Disgust (100)	Sadness
**Toxic Food Environment**	Disgust	3.8	3.3	4	Disgust (50), Neutral (38), Sadness (12)	Sadness
**Custard**	Disgust	3.2	5.3	4.0	Disgust(78), Amusement(22)	Disgust
**Rotting meat**	Disgust	3.9	4.6	4.7	Disgust (56) Neutral (33) Amuse(11)	Unsettled, disgust
**Rotting Chicken**	Disgust	3.6	4.4	4.4	Disgust (75), Neutral (25)	Disgust
**WWF T-Shirt**	Guilt	4.4	4.3	4.9	Neutral(29), Guilt(29), Shame (14), Pride(7), Fear(7), Happy(7), Anger(7)	Guilt, compelled
**WWF Laptop**	Guilt	4.4	3.7	5.0	Neutral(45), Guilt (33), Anger(11), Contempt(11)	Shame

**Don't Almost Give – Jack**	Guilt	4.2	3.4	5.2	Sadness(67), Neutral(22), Contempt (11)	Guilt, shame
**Strawberry Wasted**	Guilt	4.1	4.1	4.4	Neutral (37), Guilt(29), Sadness(14), Amusement (14), Anger(7)	Amusement, contempt, guilt, sadness, shame
**60 Seconds Blood Donation**	Guilt	5.3	3.5	5.5	Neutral (37), Guilt (25), Pride (25), Sadness (13)	Happiness, sadness
**Without…**	Guilt	3.5	4	4.1	Sadness (57), Guilt(22), Neutral(14), Anger(7)	Guilt, sad, shame
**Let Them Figure It Out**	Guilt	4.9	3.4	4.7	Guilt(23), Neutral(22), Anger(11), Happy(11), Sad(11), Fear(11), Shame(11)	Neutral, contemplative
**Black Friday**	Guilt	3.8	4.0	4.2	Neutral(44), Sadness(33), Guilt(12), Shame(11)	None
**One Child**	Guilt	2.4	4.8	3.4	Sadness(64), Guilt(22) Anger(7), Shame(7)	Guilt, shame, sad
**Shoeboxes**	Guilt	2.7	5.1	3.3	Guilt(34) Sadness(22), Anger(11) Neutral(11) Disgust(11) Fear(11)	Sadness, guilt
**Playing Cards**	Neutral	5.0	2.6	5.1	Neutral(89), Amusement(11)	Curious
**Stickers**	Neutral	5.1	2.8	5.0	Neutral(89), Amusement(11)	None
**Rubber Gloves**	Neutral	5.1	2.3	4.6	Neutral (100)	Curious
**Fibre Optics**	Neutral	5.6	2.8	5.1	Neutral(89), Happiness(11)	Curious
**Erasers**	Neutral	5.3	2.5	5.1	Neutral(75), Happy (12) Amused (12)	Curious
**Cotton**	Neutral	5.2	3.0	5.1	Neutral(78), Amusement(22)	None
**I am Canadian**	Pride	6.9	5.5	6.5	Pride(50), Amusement(29), Neutral(14), Happy(7)	Amusement, annoyance, happy
**The Anthem**	Pride	7.2	6.0	6.6	Pride(72), Happy(21), Neutral(7)	Contempt, connection, happy
**Made From Canada**	Pride	6.5	5.1	5.6	Pride(57), Neutral(22), Happy(14), Amusement(7)	Happiness

**Greatest Human Achievements**	Pride	6.2	3.1	5.4	Neutral(43), Pride(43), Fear(7), Happy (7)	Pride, tranquility, curiosity
**Peacekeeping**	Pride	5.6	3.1	5.1	Neutral(38), Happy(25), Pride(25), Amusement(12)	None
**Fly the Flag**	Pride	6.1	4.1	5.6	Neutral(45) Happy(22) Contempt(11) Amusement(11), Pride(11)	Happiness
**Last Minutes with Oden**	Sadness	2.2	5.8	3.9	Sadness (100)	Tearfulness
**Notification**	Sadness	3.0	4.3	3.3	Sadness (100)	Amusement
**Madly in love**	Sadness	3.8	4.7	3.9	Sadness (100)	Fear, happiness
**Dying Wife**	Sadness	4.6	6.1	4.1	Sadness(88), Fear(12)	Fear, love, happiness
**SickKids vs Cancer**	Sadness	2.2	3.9	4.1	Sadness(86), Neutral(7), Pride(7)	Sadness, despair, hopefulness, urgency, happiness, pride
**12 Days of Christmas**	Sadness	2.6	5.8	4.4	Sadness(76), Neutral(12) Shame(12)	Sadness, fear

Supplemental Table 3. Video clips

Video	Emotion	Link to video
60 Seconds Blood Donation§	Guilt	https://youtu.be/Y2A1xfWztBk
Africare – Shoeboxes*~	Guilt	https://youtu.be/3DYodeY8VPU
Black Friday=	Guilt	https://youtu.be/a9MRVesxS0U
WWF Laptop†	Guilt	https://youtu.be/R6QUkB-CGqE
WWF T-Shirt†	Guilt	https://youtu.be/yvR3KbEI_Ck
Don't Almost Give-Jack+=	Guilt	https://youtu.be/9ONRp5sXYus
Let Them Figure It Out†	Guilt	https://youtu.be/OKGZseeD2aY
One Child*§=	Guilt	https://youtu.be/L-QJKW3lNZA
Strawberry Wasted	Guilt	https://youtu.be/Hj7m5ZSv8Mo
Without... =	Guilt	https://youtu.be/W_0kIULLt_I
Go First	Amusement	https://youtu.be/bZjGSZ8qBbc
Dad Makes Cookies	Amusement	https://youtu.be/SRaWdYA8tZk
Charlie Sheen	Amusement	https://youtu.be/CoViV_3j6uQ
French	Amusement	https://youtu.be/xW0Ake3QDgY
Haircut	Amusement	https://youtu.be/6CenKSgHxms
Sinking	Amusement	https://youtu.be/80kM27CNqaA
Drinking 6000 Blended Maggots°•	Disgust	https://youtu.be/YoySj8QTV88
Custard‡	Disgust	https://youtu.be/mgrKM-IK9s0
The Poop Lady‡	Disgust	https://youtu.be/2DAIMvPODmI
Rotting Chicken	Disgust	https://youtu.be/DtleFGNN6vc
Rotting Meat	Disgust	https://youtu.be/3cdeD8jTBzI
Toxic Food Environment•	Disgust	https://youtu.be/V4eszz_Q9cA
Cotton	Neutral	https://youtu.be/A5u_RiiWjSs
Erasers	Neutral	https://youtu.be/HGmd2UFbc0w
Fibre Optics	Neutral	https://youtu.be/5OJOT37eEA4
Playing Cards	Neutral	https://youtu.be/sz0SmZCJty0
Rubber Gloves	Neutral	https://youtu.be/5SpTxT8fm6I
Stickers	Neutral	https://youtu.be/Xha6OwdU_L8
Fly the Flag	Pride	https://youtu.be/pB6HFRq2Ljo
Greatest Human Achievements	Pride	https://youtu.be/zG4hd5SFfSM
I Am Canadian	Pride	https://youtu.be/7g6P4GbXivQ
Made From Canada	Pride	https://youtu.be/nCh4BV9uwik
Peacekeeping~	Pride	https://youtu.be/dKJ5Hv-ClRY
The Anthem	Pride	https://youtu.be/PEQu4OHDYAQ
12 Days of Christmas+§	Sadness	https://youtu.be/XRCKtX1rDuQ
Dying Wife+§	Sadness	https://youtu.be/UDdD5UoIMxg
Madly in love§	Sadness	https://youtu.be/sHPUikEBL-0
Last Minutes with Oden°	Sadness	https://youtu.be/UDdD5UoIMxg

Notification+~	Sadness	https://youtu.be/BgesIZPfD9M
SickKids vs Cancer§*	Sadness	https://youtu.be/sHPUikEBL-0

Content warnings:
§Serious illness, injury, hospital
* Child death
~War
=Poverty/homelessness
†Climate change
+Death
•Vomiting
°Animal death
‡Consumption of body waste

Supplemental Table 4. Feedback Statements

Video	Statement
Black Friday	You like to have new things
One Child	You donate less than the average Canadian to international relief efforts
Don't Almost Give- Jack	You could do more to help people in Canada
Africare-Shoeboxes	You don't want to be repeatedly asked to donate money
Without...	You sometimes ignore charity appeals
Strawberry Wasted	You waste much more food than average
WWF Laptop	You waste more energy than average
WWF T-Shirt	Your laundry habits waste more water than two thirds of respondents
60 Seconds Blood Donation	You think blood and organ donation is as important as most Canadians
French	You find humourous ads more memorable than the average Canadian
Sinking	You can always learn a new language
Charlie Sheen	You are as influenced by celebrity endorsement as most Canadians
Dad Makes Cookies	You bake less than the average Canadian
Go First	You are as influenced by ads as most people
Haircut	Your opinion of adoption is shared by half of Canadians
Rotting Chicken	You worry about rotted food as much as the average person
Drinking 6000 Blended Maggots	You feel as sick about maggots as the average person
Rotting Meat	You would never eat rotted meat
The Poop Lady	You should not eat contaminated food
Toxic Food Environment	You are as anxious about vomiting as the average person
Custard	You would not eat food contaminated with bodily fluids

Cotton	You will see a video about cotton thread
Rubber Gloves	You will see a video about rubber gloves
Fibre Optics	You will see a video about fibre optics
Erasers	You will see a video about erasers
Playing Cards	You will see a video about playing cards
Stickers	You will see a video about stickets
I Am Canadian	You are in Canada
Made From Canada	Your sense of Canadian identity is as strong as average
Anthem	Your sense of connection to the world is stronger than average
Peacekeeping	You think peacekeeping is as important as most Canadians
Fly the Flag	You feel connected to Canada
Greatest Human Achievements	You are proud of humanity's achievements
Dying Wife	You get sad about as easily as other people
SickKids vs Cancer	You think fighting cancer is important
Last Minutes with Oden	You feel sorry for people who have recently lost a pet
12 Days of Christmas	You should not drive unsafely
Notification	You feel sorry for people who have recently lost a child
Madly in love	You feel sad when someone else is sad about as often as other people

Supplemental Table 5. Means, standard deviations, and ranges for trait and state characteristics. 77 participants (36 women, 41 men) completed the Guilt Inventory and EQ; 55 participants (18 women, 37 men) completed the STAI.

	**M**	**SD**	**Range**
Guilt Inventory	129.104	22.664	75-190
Empathy Quotient	44.740	10.462	21-73
State-Trait Anxiety Inventory			
State	28.815	7.8333	20-55
Trait	30.259	11.818	20-74

Supplemental Table 6. Total counts of primary emotions endorsed by target emotion

Target Emotion / Endorsed Emotion	Amusement	Disgust	Guilt	Neutral	Pride	Sadness
Amusement	**372**	46	12	87	56	2
Anger	2	2	45	1	3	11
Contempt	6	5	25	6	6	4
Disgust	1	**445**	17	1	0	1
Embarrassment	3	7	45	0	2	0
Fear	3	4	27	1	1	7
Guilt	2	1	**222**	0	1	9
Happiness	65	1	14	27	87	26
Neutral	103	44	147	**435**	92	30
Pride	5	0	22	6	**314**	2
Sadness	2	8	266	0	2	**470**
Shame	0	1	80	0	0	2

Supplemental Table 7. Total numbers of primary emotions endorsed and congruence with the video's target emotion, with total emotions broken down by valence and social nature

	Amusement	Disgust	Guilt	Neutral	Pride	Sadness
Total Emotion Endorsements	564	564	922	564	564	564
Total Non-target Emotions Endorsed	192	119	700	129	250	94
Average Target Accuracy	65.957%	78.901%	24.078%	77.128%	55.674%	83.333%
Total Positive Emotions	442	47	48	120	401	30
Total Negative Emotions	19	473	727	9	15	504
Total Negative Social Emotions	5	9	347	0	3	11
Total Positive Social Emotions	5	0	22	6	314	2

Supplemental Table 8. Counts of secondary emotions endorsed by target emotion

Target Emotion / Endorsed Emotion	Amusement	Disgust	Guilt	Neutral	Pride	Sadness
Amusement	**127**	67	35	84	112	13
Anger	9	66	125	3	2	69
Contempt	11	73	65	12	22	18
Disgust	3	**149**	56	3	1	11
Embarrassment	19	41	128	1	7	13
Fear	12	62	70	3	7	90
Guilt	12	13	**306**	5	3	59
Happiness	177	8	36	43	301	77
Neutral	132	54	97	**182**	70	57
Pride	33	0	25	16	**185**	25
Sad	13	45	229	3	13	**135**
Shame	8	28	281	1	2	29

Supplemental Table 9. Total numbers of secondary emotions endorsed, with total emotions broken down by valence and social nature

	Amusement	Disgust	Guilt	Neutral	Pride	Sadness
Total Secondary Emotions Endorsed	556	606	1453	356	725	596
Total Non-target Emotions	429	457	1147	174	540	461
Total Positive Emotions	337	75	96	143	598	115
Total Negative Emotions	87	477	1260	31	57	424
Total Negative Social Emotions	39	82	715	7	12	101
Total Positive Social Emotions	33	0	25	16	185	25

Supplemental Table 10. Results of paired Wilcoxon tests for AU 4-Brow Lowerer comparing amusement (M=16.91), disgust (M=26.98), neutral (M=19.51), pride (M=14.12), and sadness(M=19.90). *p≤0.05, **p≤0.01, ***p≤0.001

	Amusement	Disgust	Neutral	Pride
Disgust	735***			
Neutral	1083	1942**		
Pride	1670*	2211***	1925***	
Sadness	1068	1900**	1392	703***

Supplemental Table 11. Results of paired Wilcoxon tests for AU 12-Lip corner puller comparing amusement (M=19.18), disgust (M=16.45), neutral (M=5.65), pride (M=6.98), and sadness (M=8.68) *p≤0.05, **p≤0.01, ***p≤0.001

	Amusement	Disgust	Neutral	Pride
Disgust	1807.5*			
Neutral	2484***	2114.5***		
Pride	2300***	2087***	1170	
Sadness	2299.5***	1889**	918	912

Supplemental Table 12. Results of paired Wilcoxon tests for head tilt down comparing amusement (M=11.60), disgust (M=21.17), neutral (M=9.29), pride (M=15.02), and sadness (M=11.23) *p≤0.05, **p≤0.01, ***p≤0.001

	Amusement	Disgust	Neutral	Pride
Disgust	503.5***			
Neutral	1191	1612.5***		
Pride	803	1116*	752	
Sadness	1062	1598***	683	935

Supplemental Table 13. Results of paired Wilcoxon tests for head turn comparing amusement (M=7.67), disgust (M=19.05), neutral (M=6.82), pride (M=9.10), and sadness (M=8.54) *p≤0.05, **p≤0.01, ***p≤0.001

	Amusement	Disgust	Neutral	Pride
Disgust	347.5***			
Neutral	757.5	1597 ***		
Pride	609.5	1209 ***	512.5	
Sadness	709	1584.5***	557	591

Supplemental Table 14. Results of paired Wilcoxon tests for face touch comparing amusement (M=7.99), disgust (M=10.04), neutral (M=7.78), pride (M=8.33), and sadness (M=6.80) *p≤0.05, **p≤0.01, ***p≤0.001

	**Amusement**	**Disgust**	**Neutral**	**Pride**
Disgust	456.5			
Neutral	634.5	574.5		
Pride	479.5	539.5	533	
Sadness	739	771	546	525.5
